# Kea (*Nestor notabilis*) represent object trajectory and identity

**DOI:** 10.1038/s41598-019-56380-4

**Published:** 2019-12-24

**Authors:** Amalia P. M. Bastos, Alex H. Taylor

**Affiliations:** School of Psychology, The University of Auckland, Private Bag 92019, Auckland, 1142 New Zealand

**Keywords:** Psychology, Animal behaviour

## Abstract

The ability to represent both the identity and trajectory of hidden objects underlies our capacity to reason about causal mechanisms. However, to date no studies have shown that non-human animals are capable of representing these two factors simultaneously. Here, we tested whether kea can represent out-of-sight object trajectories and identities by presenting subjects with three tasks, each of which involved tracking or predicting hand trajectories as they moved behind a screen. Taken together, our results suggest that kea have the capacity for mental simulation in complex tasks involving moving hidden objects.

## Introduction

Humans are capable of representing both the trajectory and identity of moving out-of-sight objects^1–4^. For example, if we see a bird fly behind a tree, we expect the same bird to emerge from behind the tree on the same trajectory. This ability is a key foundation underlying our ability to simulate complex out-of-sight interactions, including hidden causal mechanisms^5,6^.

Research on non-human animals has largely focused on object trajectory, using invisible displacement and spatial transposition tasks, rather than the combination of object trajectory and identity. In invisible displacement tasks, an object is placed in a displacement device, and this device is then moved behind a screen. The device is then shown to be empty. This requires subjects to infer, based on the observed trajectory of the device, that the object must have been left behind the screen. In spatial transposition tasks, an object is placed under one of several displacement devices, which are moved to different positions. Subjects have to track the visible trajectory of the device containing the object. These tasks have been solved by children^7–9^, corvids^10–12^, psittacines^13–17^, and great apes^18–21^, although it remains unclear whether any species other than humans and the great apes have succeeded in these tasks using mental representation, rather than simpler associative learning strategies^20,22^; but see^23^. Nevertheless, there is convincing evidence that several species, including dogs, monkeys, parrots, corvids, and chickens can represent object identity in other tasks^13,24–31^, and chickens have also been shown to represent trajectory, correctly predicting the end-destination of an object behind one of two screens^32–34^.

So far, none of these tasks have required an animal to simultaneously represent both the hidden trajectories of multiple objects, and the identity of these objects, as humans can^1^. Kea (*Nestor notabilis*) are an ideal candidate species for examining this possibility, due to their sophisticated sensorimotor intelligence^35,36^. Here, we tested whether kea could simultaneously represent the trajectory and identity of objects, using tasks where subjects had to represent the identity of hidden tokens while making predictions about their trajectories.

## Results

### Experiment 1

**Figure 1:**
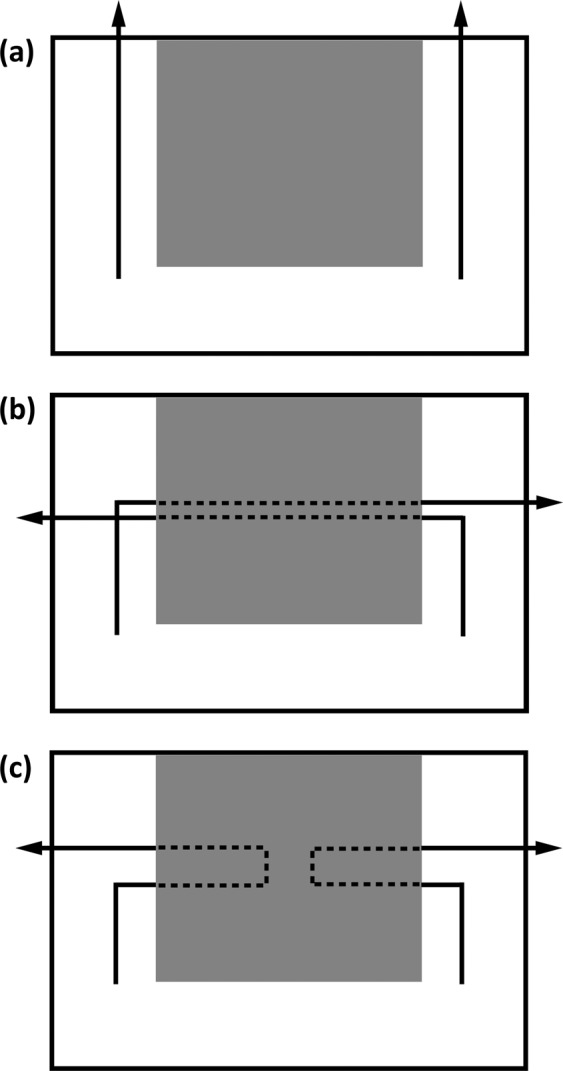
Experiment 1 (**a**) parallel, (**b**) crossed, and (**c**) split trajectories of Experiment 1. The area covered by the occluding screen is shown in grey. Solid lines indicate the visible parts of the trajectory, and dotted lines indicate the paths followed by the hands behind the occluder.

Experiment 1 used three conditions to test if kea could predict hand trajectories after they moved behind a screen, based only on their initial movement. In all conditions, trials began when the experimenter demonstrated that one of their hands held a rewarding black token and the other an unrewarding orange token. Kea then observed the hands either moving parallel to a cardboard occluder (approximately 20 cm × 20 cm) attached to a plexiglass screen (43 cm × 29 cm) in the parallel trials, or behind the occluder, in crossed and split trials (Fig. 1). In crossed trials, the experimenter’s hand moved towards the occluder from opposite sides, crossed behind the occluder, and then emerged on the opposite sides. In split trials, the hands moved identically at the start of the trial, but after moving behind the occluder, instead of crossing over, they were returned to their initial starting positions. Parallel trials served as a control condition, as throughout these trials both hands were visible at all times. They also ensured subjects were motivated to carry out testing, increasing the number of solvable trials in each block. At the end of each trial, kea were allowed to choose one of the hands to indicate where they believed the rewarding token must be. Ten kea experienced up to two sets of sixty trials, presented over the course of multiple testing sessions. Only fifteen trials were presented in each testing session, consisting of five trials for each of the three trajectory types (crossed, parallel, and split trajectories).

If subjects were predicting hand trajectories behind the screen from their initial movement, we expected them to perform above chance at the crossed trials, and at or below chance for split trials. This pattern could not be explained by subjects using hand identity or simple associative rules, such as searching the location on the same side as where the token was first picked up. It also ruled out the use of unintentional cues provided by the experimenter on the location of the token, given that in all trial types, the experimenter knew in which hand the rewarding token was located. Subjects that succeeded in parallel and crossed trial types, but not split trial types, within either their first or second set of sixty trials (20 trials of each trajectory type), proceeded to Experiment 2. Subjects that succeeded in parallel and split trials, but not crossed trials, did not participate in Experiment 2, and instead proceeded to Experiment 3. All subjects were given up to two sets of sixty trials to learn the contingencies of the tasks.

Four of the ten kea tested showed the pattern of results predicted if kea were able to represent object trajectory and identity, performing better than chance in parallel trials and crossed trials and at chance in the split trials (two-tailed Bayesian binomial tests, relative preference 0.5, BF > 3, Table 1). Whilst two subjects (Bruce and Cheeky) achieved this within their first set of trials, two other subjects (Loki and Neo) succeeded in their second set of trials, suggesting that they took some time to learn the affordances of the task. In contrast, three subjects (Harley Quinn, Moriarty, and Spike) appeared to adopt a strategy relying on the proximity of the two hands to the trajectory starting points to make their choices, as they performed better than chance in split but not crossed trajectory trials. No subjects performed above chance in both split and crossed conditions, suggesting that they did not use hand identity or unintentional cues by the experimenter to guide their choices.

**Table 1:** Performance of all ten kea in Experiment 1, followed by performance of the four subjects in Experiment 2.

Subject	Experiment 1		Experiment 2
	Performance in First 60 Trials (Number of times black token was chosen)	Performance in Following 60 Trials (Number of times black token was chosen)	Performance over 40 Trials (Number of correct choices)
Blofeld	Parallel: **20/20**	Parallel: **20/20**	NA
	Split: 10/20	Split: 12/20	
	Crossed: 10/20	Crossed: 10/20	
Bruce	*Parallel*: **20/20**	NA	Hidden: **9/20** Visible: **20/20**
	*Split*: **5/20**		
	*Crossed*: **19/20**		
Harley Quinn	Parallel: **19/20**	NA	NA
	Split: **20/20**		
	Crossed: 6/20		
Cheeky	*Parallel*: **19/20**	NA	*Hidden*: **17/20** *Visible*: **18/20**
	*Split*: **3/20**		
	*Crossed*: **16/20**		
Loki	*Parallel*: **20/20**	*Parallel*: **20/20**	*Hidden*: **19/20** *Visible*: **20/20**
	*Split*: 9/20	*Split*: 8/20	
	*Crossed*: 12/20	*Crossed*: **19/20**	
Moriarty	Parallel: **17/20**	Parallel: **15/20**	NA
	Split: 14/20	Split: **19/20**	
	Crossed: 9/20	Crossed: 9/20	
Neo	*Parallel*: **19/20**	*Parallel*: **20/20**	*Hidden*: **20/20** *Visible*: **20/20**
	*Split*: 6/20	*Split*: **5/20**	
	*Crossed*: 14/20	*Crossed*: **19/20**	
Plankton	Parallel: **18/20**	Parallel: **20/20**	NA
	Split: 13/20	Split: 14/20	
	Crossed: 8/20	Crossed: 7/20	
Taz	Parallel: **20/20**	Parallel: **20/20**	NA
	Split: 11/20	Split: 10/20	
	Crossed: 11/20	Crossed: 11/20	
Spike	Parallel: **19/20**	NA	NA
	Split: **18/20**		
	Crossed: **3/20**		

Subjects highlighted in italics showed performances consistent with using a mental representation strategy to solve the tasks within each of the two experiments. Bold performances denote Bayes Factor >3. Full data for each set of trials, categorised by trajectory type is provided in Table S2.

### Experiment 2

Experiment 2 tested whether the four successful kea from Experiment 1 had succeeded by using an associative strategy: when hands move towards the middle of the screen, switch sides. If kea followed this rule they should make a specific error: after seeing a hand move towards the middle of the screen they should then choose the hand on the opposite side of the screen, even if they had previously seen that this hand was holding the non-rewarding token. In contrast, if kea represented the hand containing the rewarding token as it moved behind the screen, then we expected them to be able to ignore the hand containing the non-rewarding token.

Subjects were given two blocks of 20 trials where one hand moved behind, and followed the trajectory of, an opaque U-shaped piece of cardboard behind the plexiglass screen, whilst the other hand visibly moved towards the opposite end of the U-shape, and then away to the other side of the plexiglass, as if continuing the motion of the hidden hand (Fig. 1). The side tokens were placed on, the side the U-shape was positioned on, and whether trajectories for the rewarding black token were visible or hidden (behind the U-shape) were counterbalanced within blocks. Subjects experienced 20 visible trajectory trials, and 20 hidden trajectory trials.

Of the four kea tested, three performed above chance within their first 20 trials of both visible and hidden trajectory conditions (two-tailed Bayesian binomial tests, relative preference 0.5, BF > 3, Table 1), indicating these kea were not using the associative rule of searching in the location opposite to where the token was first picked up.

### Experiment 3

Experiment 3 expanded on the results from Experiments 1 and 2, and was designed to test whether kea are capable of predicting the trajectory of a single token, rather than guessing their likely trajectory after both hands were presented. Unlike the previous experiments, this involved only a single trajectory carried out by one hand, and the kea never observed its end-destination until they had made a choice. Besides testing for kea’s ability to predict trajectories given no information of their potential end-points, this also removed any hand-identity or timing-associated cues that might have been present from tasks using two simultaneously moving hands. Therefore, this experiment was designed to confirm that kea are capable of representing novel trajectories and had not relied on these associative rules to succeed in the two previous experiments.

**Figure 2:**
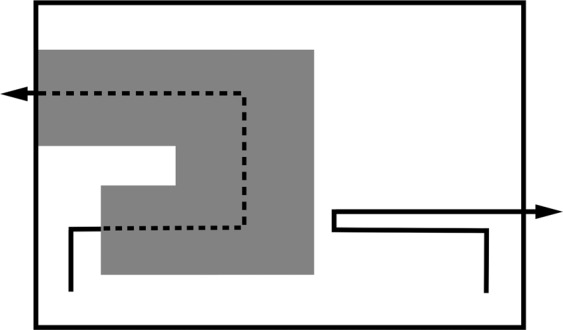
The two trajectories for Experiment 2, with one hand moved behind the U-shaped screen (hidden trajectory), and the other moved simultaneously on the opposite side of the screen (visible trajectory). Solid lines indicate the visible parts of the trajectory, and dotted lines indicate the paths followed by the hands behind the occluder, represented by a grey rectangle.

Kea were first trained to search behind two windows (∅6 cm, 21 cm apart) on a large plexiglass screen (45 cm × 45 cm) in order to find food, but were not given any information on potential hidden trajectories that the hands might take. At test, kea had to predict which of two windows in a screen they should look behind to find a hand containing a token, after observing the hand moving toward**s** the screen along one of four different paths, and then following an occluded trajectory (Fig. 2). Kea were presented with 20 trials of each trajectory type (top horizontal, bottom horizontal, top-bottom diagonal, bottom-top diagonal; Fig. 2). The presentation of the four trajectory types was pseudorandomised and counterbalanced within blocks of 20 trials. Testing was carried out by an experimenter blind to hypotheses, wearing mirrored sunglasses. Seven kea were presented with Experiment 3 (one kea had been transferred to another facility and two kea subjects did not habituate to the new apparatus).

**Figure 3:**
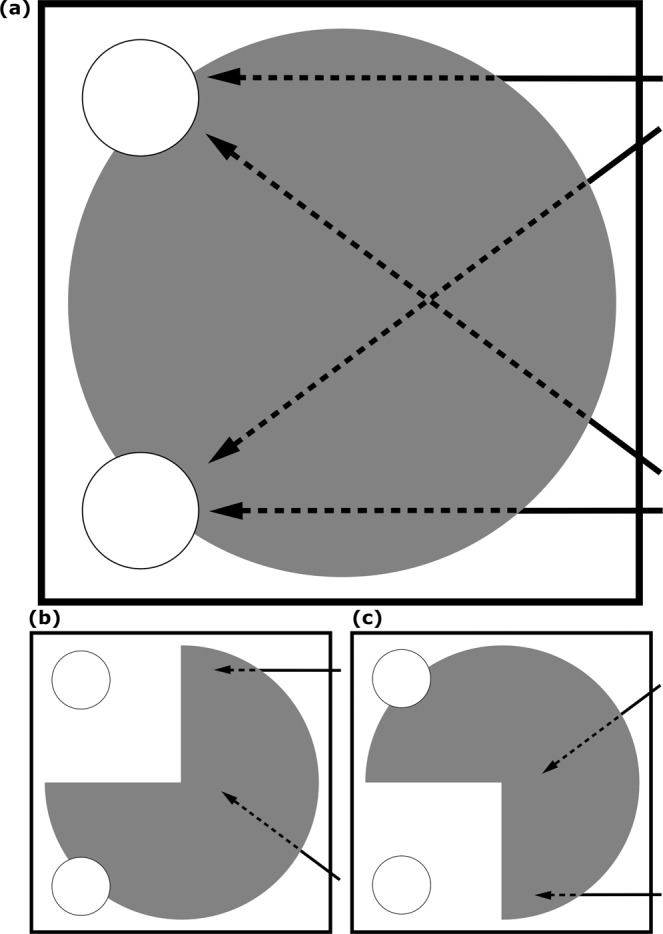
Conditions of Experiment 3. In (**a**), we show the four trajectory trial types in Experiment 3, from top to bottom: top horizontal, top-bottom diagonal, bottom-top diagonal, bottom horizontal. Parts (**b**,**c**) illustrate the two possible set-ups for Experiment 3’s control conditions, showing the four trajectories: top horizontal, bottom-top diagonal, top-bottom diagonal, bottom horizontal. In all images, the area covered by the occluding screen is shown in grey. Solid lines indicate the visible parts of the trajectory, and dotted lines indicate the trajectories kea were expected to predict, given the hand’s initial movement.

A control condition was then performed where we tested whether subjects would flexibly change their prediction of an object’s trajectory when given contradictory information. Subjects experienced trials where the experimenter’s hand appeared to start on one of the four previously-seen trajectories before becoming occluded, but this time part of the occluding barrier was removed, suggesting that the hand could not have reached its expected destination (Fig. 3). Subjects had to realise that, given that the previously-rewarded trajectory was no longer possible, the experimenter’s hand must have followed an alternate path and ended at the opposite window, flexibly adjusting their predictions of these trajectories given this novel information.

Four of seven subjects performed better than chance within their first 20 trajectory prediction trials (two-tailed Bayesian binomial tests, relative preference 0.5, BF > 3, Table 2). Their performance shows these kea readily represented and predicted the end-destination of four novel object trajectories. Interestingly, a subject that had previously failed to pass Experiment 1 (Moriarty), was successful in Experiment 3, suggesting that predicting a single trajectory might be less cognitively taxing than simultaneously representing two object identities and their trajectories. All seven subjects succeeded within their first 20 control trials, showing that they flexibly updated their predictions when they were given information to suggest that the previously-rewarding trajectory had become impossible.

**Table 2:** Performance of seven kea in Experiment 3.

Subject	Trajectory Prediction Trials: Performance in First 20 Trials (Number of times black token was chosen)	Control Condition Trials: Performance in First 20 Trials (Number of times black token was chosen)
Blofeld	14/20	**18/20**
*Bruce*	***17/20***	***18/20***
*Loki*	***16/20***	***19/20***
*Moriarty*	***16/20***	***19/20***
*Neo*	***17/20***	***17/20***
Plankton	13/20	**16/20**
Taz	10/20	**20/20**

Subjects highlighted in italics showed performance consistent with predicting the end-point of a novel trajectory. Bold performances denote Bayes Factor >3. Full data for each set of trials categorised by trajectory type is provided in Table S3.

Subjects’ performance was not stable over time: only one subject, Blofeld, consistently improved in their performance over the course of 80 trials, whilst it decreased over the course of multiple trials for Loki and Moriarty, who performed no differently from chance in their final block of 20 trials (Table S3).

We also analysed subject performance in terms of the number of times they selected the window closest to the last location they last saw the experimenter’s hand. We found no evidence to suggest that any subjects were using this proximity cue to make their choices, a hypothesis that would have predicted above-chance selection of the top window for both top horizontal and top-bottom trajectories, and selection of the bottom window for bottom horizontal and bottom-top trajectories (two-tailed Bayesian binomial tests, relative preference 0.5, Table 3). However, this analysis revealed a clear bias for the bottom window in one subject, Plankton (two-tailed Bayesian binomial tests, relative preference 0.5, BF > 3, Table 3).

**Table 3:** Kea’s choices of window most closely located to last place hand was seen (use of a proximity cue).

Subject	Trajectory Prediction Trials	
	Proximity Top	Proximity Bottom
Blofeld	16/40	21/40
Bruce	18/40	22/40
Loki	**9/40**	25/40
Moriarty	14/40	19/40
Neo	16/40	22/40
Plankton	**8/40**	**32/40**
Taz	26/40	19/40

No subjects showed performance consistent with using this proximity cue. Bold performances denote Bayes Factor >3. Full data for each set of trials categorised by trajectory type is provided in Table S3.

## Discussion

Our study shows that kea are capable of simultaneously remembering the identity of two hidden tokens and representing out-of-sight trajectories. In Experiment 1, four out of ten kea tested identified the end location of a preferred token when it was hidden in a hand and then moved behind a screen along a specific trajectory. However, they either selected the wrong hiding location, or performed at chance, when the trajectory was surreptitiously changed behind a screen, suggesting that they were tracking hand trajectories. Experiment 2 showed three of these four kea were not simply following an associative rule where they picked the hand that appeared on the opposite side of the occluder after moving behind it. In Experiment 3, four out of seven kea tested were able to predict the end-point of four novel trajectories within their first twenty trials, and then flexibly adjust their choices when these trajectories became impossible.

Taken together, these results demonstrate that kea have the capacity to represent the trajectories and identities of hidden objects simultaneously, as well as predict the end-points of novel trajectories. Three subjects performed above chance in crossed and parallel trials for Experiment 1, and in visible and hidden trials for Experiment 2, as would be predicted if they were using a mental representation strategy to solve both tasks. One of these three subjects (Cheeky) was transferred to another facility prior to Experiment 3, but the remaining subjects (Neo and Loki) also passed Experiment 3. Kea’s performance across these three experiments suggests that they have the capacity to simulate out-of-sight trajectories, and that the successful subjects did not rely on unintentional experimenter cues, hand identities, or simple associative strategies to succeed in these tasks.

However, the variation between performances in our sample of kea shows that although kea, as a species, have the cognitive capacity to represent object identity and trajectory simultaneously, some do not spontaneously adopt this as a primary strategy in such tasks. Two individuals clearly searched in the last location they saw a hand in Experiment 1, irrespective of its trajectory. One individual used a strategy of searching opposite where they last saw a hand, as evidenced by their failure in Experiment 2. This suggests that although mental simulation is within the realm of kea’s cognitive abilities, it is not always their preferred strategy to solve problems involving out-of-sight objects. Further research is required to establish exactly under which contexts mental simulation becomes more or less likely to emerge as the preferred strategy in kea, and other nonhuman animals, and to further investigate performance at both the individual and group levels.

There was also variation in performance within individuals over the course of eighty trajectory prediction trials for Experiment 3. A large number of trials may have proven repetitive and therefore frustrating for the kea, leading to reduced performance over time. Similarly, kea may have stopped attending as closely to the task after experiencing a large number of trials on the same problem. This decline in performance was not observed in the control conditions, possibly due to the easier nature of this task, which may have elicited less frustration when repeated over a large number of trials. It is also possible that kea selected the only likely option in the control condition before the token began on its trajectory, realising that this was the only possible solution to the problem without having to reason about possible trajectories. However, as kea were required to attend to the visible trajectory prior to making a choice in our experiments, they were not given the opportunity to show anticipatory behaviour, that is, select the only possible choice before the trajectory began. Further work will be required to distinguish between these two strategies. Nevertheless, four subjects’ above chance performance within their first twenty trials of the trajectory prediction conditions in Experiment 3 supports the claim that kea can make predictions about trajectories’ end-destinations, regardless of which strategy kea used to solve the control condition.

Past work on chickens, psittacines and corvids have shown that birds can track the trajectory of a single object^11,17,32–34^, but these studies have not simultaneously tested an ability to track hidden moving objects and represent their identities. Our results provide the first evidence that kea can simultaneously represent the identities of two objects and their trajectories. These abilities are essential to understanding more complex causal mechanisms that occur partially or entirely out-of-sight^5,6^. This research therefore opens up the possibility of exploring how much kea, and other animals, understand the complex causal mechanisms of the world around them.

## Material and Methods

### Ethics statement

This research was conducted under ethics approval from The University of Auckland Ethics Committee (reference number 001816). Our research was conducted in accordance with the New Zealand National Animals Ethics Advisory Committee guidelines.

### Subjects

We tested ten kea, housed in a large outdoor aviary in Willowbank Wildlife Reserve (see Table S1 for subject details). Food and water were available *ad libitum* within the aviary. All participation was voluntary and subjects were free to leave the testing platform at any time.

### Materials

Subjects were tested on individual training platforms (42 cm × 42 cm) within the aviary and rewarded with Hill’s Science Diet pellets during trials. For training and testing, a small wooden shelf (60 cm × 20 cm) was used with a plexiglass screen separating the kea from the experimenter.

### Training: Hand Tracking Training Protocol

Subjects were trained to exchange black tokens for a reward, whereas orange tokens were unrewarding. Kea were then trained to track human hands which moved behind a fully transparent plexiglass screen over the course of three training stages.

All training stages were provided in blocks of 10 or 20 trials, using the testing apparatus. All trials began with subjects being shown two empty hands facing them, which then performed different actions in turn. Once the hands had reached their final positions at the top of the screen as closed fists, subjects made a choice by touching their beak or cere to one of the experimenter’s fists. Only one choice was allowed per trial. In trials where a black token was chosen, the token was handed to the subject, which then had to hand it back to the experimenter in exchange for a pellet. Where the subject made the incorrect choice of picking an empty hand or a hand containing an orange token, the contents of the hand were shown and then the hand was retracted, and the next trial was started immediately. Criterion is specified for each training stage in parentheses, below:

**Stage 1:** Kea must choose the correct hand when one hand holds a pellet, and one hand is empty:

- When both hands move up simultaneously and in parallel. **(17/20 trials)**
- When two trial types are interspersed: either both hands move up simultaneously and in parallel, or when both hands are moved simultaneously and in parallel, then crossed over above the plexiglass such that they end up on the opposite sides. During this stage, subjects could touch one or both hands at multiple times as they were crossed over. **(17/20 trials)**
- When two trial types are interspersed: either both hands move up simultaneously and in parallel or when both hands are moved simultaneously and crossed over behind the plexiglass such that they end up on the opposite sides. At this stage, subjects were required attend to the motion of both hands when it occurred behind the screen, without touching either hand. **(17/20 trials)**

**Stage 2:** Kea must choose the correct hand when one hand holds the black token, and one hand is empty, in interspersing parallel and crossed trial types as in Stage 1c. This ensured that subjects remembered the black token was rewarding and still made the correct choice when the pellet was substituted for a rewarding token. **(10/10 trials)**

**Stage 3:** Kea must choose the correct hand when hands pick up, show, and then enclose either the rewarding black token, or the unrewarding orange token, in interspersed trials with parallel and crossed trajectories, as in Stages 1c and 2. In this case, subjects had to keep track of two hands holding two different objects, only one of which was rewarding. **(17/20 trials)**

### Training: Food-search Choice Task for Experiment 3

Before taking part in Experiment 3, subjects were habituated to putting their beaks through a small window (∅6 cm), and trained to make functional choices between two options in a food-searching task. These new behaviours were easy to interpret (i.e. if a subject placed their beak through a window, this was considered their final choice) and encouraged subjects to make a clear choice at test. All training steps (and subsequent testing for all subjects) were conducted by experimenters blind to hypotheses, wearing mirrored sunglasses. This was done as a precaution against Clever Hans cueing, i.e. subjects using experimenter’s unintentional cues to guide their choices in a task. All training steps required a **17/20 criterion** to be reached before proceeding to the next stage of training. They are detailed below:

1. **Touch visible fist with beak**. Subjects were habituated to standing on a wooden step and placing their beak through a small round window (∅6 cm) in the middle of a piece of plexiglass (45 cm × 25 cm) to touch the experimenter’s closed fist. The experimenter’s fist contained a Science Diet pellet which was given to the subject after they touched the fist.
2. **Search for experimenter’s hand**. In this step, the same piece of plexiglass was used, with a cardboard square (25 cm × 25 cm) on the back, surrounding the central window. Now, the experimenter placed their closed fist behind the cardboard in any position. Subjects were required to place their beak into the window and ‘search for’ the experimenter’s hand. Once their beak touched the experimenter’s fist, they were rewarded with a Science Diet pellet. This ensured that subjects were still motivated to search for the experimenter’s fist and put their beak through the window even when the hand was not visible.
3. **Search for a piece of food**. This was identical to the previous step, but the Science Diet pellet was directly attached to the back of the cardboard, so the subjects would search for it directly with their beak. This required a much longer search time, ensuring that subjects were fully comfortable placing their beaks through the window in the plexiglass screen.
  The plexiglass screen was now substituted for a larger version (45 cm × 45 cm), with two identical windows (∅6 cm) drilled into the left side. A large cardboard occluder (45 cm × 20 cm) could slide over the left side to hide the two windows. The windows were equidistant from each other (21 cm) and from the edges of the plexiglass screen (6 cm). Two identical semi-circular transparent plastic baskets (approximately 6.5 cm wide × 2 cm tall, 3 cm radius) were positioned under each of the two windows on the experimenter’s side of the plexiglass. The final two training steps were done using this new apparatus. Again, these training steps required **17/20** criterion:
4. **Making a functional choice between two options**. At the start of each trial, the experimenter slid the cardboard occluder in front of the two windows in the plexiglass. Then, they shuffled a Science Diet pellet between their hands behind their backs, enclosing it into one fist and leaving another closed fist empty. Both hands were simultaneously moved towards the two windows at the same time. One of the baskets below a window was baited, and the other was sham-baited. Both these baiting episodes occurred simultaneously. Following this, both hands slid the cardboard occluder, so the kea could see which basket contained the food pellet. Baskets and hands baited were pseudorandomised and counterbalanced within blocks of 10 trials. During this step, hand motions for both hands were identical so as to avoid teaching the subjects any additional information on hand-trajectories. Subjects simply had to look into both baskets and place their beak through the correct window to obtain the food reward. Only their first choice was allowed. If subjects placed their beak through the wrong window, the cardboard occluder was immediately slid back to the left and subjects were not allowed to change their choice.
5. **Remembering the location of hidden food**. This step was identical to the last, but an additional piece of plexiglass (25 cm × 20 cm) was placed behind the cardboard. Instead of allowing the subjects to take the piece of food from the basket immediately once the cardboard occluder was slid to the right, this plexiglass was left in place. Subjects could therefore see, but not retrieve, the Science Diet pellet in one of the two baskets. After subjects had seen the Science Diet pellet, the windows were occluded by the cardboard again. The experimenter then placed two small cardboard rectangles (6.5 cm × 2 cm) into each basket simultaneously, so the pellet would no longer be visible to the subject. Both the plexiglass and cardboard occluder were now slid back to the right, and the subject was allowed to place their beak through their chosen window and remove the cardboard rectangle. If they made the correct choice, this would reveal the hidden Science Diet pellet, which they were allowed to take. If they made the wrong choice, this would reveal an empty basket, which would be immediately covered up again, and the subject would not receive a reward.

During the fourth training step, two subjects (Harley and Spike) would leave the working platform each time the large plexiglass screen was introduced. We interpreted that as a sign of fear and excluded these subjects from any further testing with this apparatus, in compliance with our lab’s code of conduct and ethics protocols. Participation in all experiments is voluntary and subjects are free to leave the platform if they do not wish to work at any given time or with a given apparatus.

### Testing Procedure

All trials began with the experimenter showing their empty hand(s) to the subject, then picking up a token and enclosing it in their fist. This was held up momentarily before the start of each trajectory. Tokens were picked up one at a time. After the trajectory was performed, the experimenter either held out both closed fists simultaneously (Experiments 1 and 2), waiting for the subject to make their choice, or hid their fist behind the occluder until the subject made a prediction about its likely trajectory (Experiment 3). Where subjects picked the incorrect hand, they were shown the unrewarding (orange) token, which was then taken away, and the next trial was started immediately. Rewarding (black) tokens were given to the subjects and subsequently exchanged for a food reward.

We took several measures to ensure kea could not use body movements as cues to identify hidden trajectories. In Experiments 1 and 2, experimenters’ shoulders were kept static, with elbows held together in the centre (so as to be fully hidden behind a rectangular occluder), and all movement was performed by the forearms. In Experiment 3, the hand appeared to follow the initial trajectory when fully visible, but always stopped half-way through, behind the occluder, rather than reaching its end destination. It only continued onto the end of the trajectory after the subject had placed their beak through the window, if they made the correct choice. Furthermore, the experimenter stood behind the large occluder (45 cm × 45 cm) in all trials, which hid their right shoulder’s movements from the subject. Experimenters also wore mirrored sunglasses so as to not provide any gaze cues to subjects.

### Analyses

All trials were coded and filmed *in situ*. Performance for every set of 20 trials of each condition were analysed at the individual level, using two-tailed Bayesian binomial tests. The test value was set at 0.5 using default beta priors. All analyses were carried out using Jasp Team 2019^37^. We followed the convention that a Bayes factor (BF) < 0.33 shows substantial support for the null hypothesis, whilst a BF > 3 shows substantial support for the competing hypothesis^38^.

## Supplementary information

Supplementary Information

## Data Availability

Our full dataset is provided in the Supplementary Information files.
