# Supplementary material for: Kea (Nestor notabilis) represent object trajectory and identity
Source: Sci Rep. 2019 Dec 24;9:19759. doi: 10.1038/s41598-019-56380-4 (PMC6930200; doi:10.1038/s41598-019-56380-4)
Supplement: Supplementary file 1 — Supplementary Information [file 41598_2019_56380_MOESM1_ESM.docx]

**Supplementary Information for ‘Kea (*Nestor notabilis*) represent object trajectory and identity’**

Amalia P. M. Bastos^1^*, Alex H. Taylor^1^

^1^ School of Psychology, The University of Auckland, Private Bag 92019, Auckland 1142, New Zealand

***** Correspondence: Amalia Bastos, a.bastos@auckland.ac.nz

**Table S1.**

| **Subject** | **Age** | **Sex** | **Origin** | **Participation (Experiments)** |
| --- | --- | --- | --- | --- |
| Blofeld | 5 | M | Captive bred | All experiments |
| Bruce | 6 | M | Wild | All experiments |
| Cheeky | 12 | M | Captive bred | Experiments 1 and 2 |
| Harley Quinn | 4 | F | Captive bred | Experiment 1 |
| Loki | 4 | M | Captive bred | All experiments |
| Moriarty | 4 | M | Captive bred | Experiments 1 and 3 |
| Neo | 6 | M | Captive bred | All experiments |
| Plankton | 4 | M | Captive bred | Experiments 1 and 3 |
| Taz | 6 | M | Captive bred | Experiments 1 and 3 |
| Spike | 23 | M | Wild | Experiment 1 |

***Table S1.*** All subjects were parent-reared. Ages (as of 2019) are estimated from hatch dates (captive bred subjects) or veterinarian evaluations (wild subjects). One further subject, Johnny, was tested but refused to complete all required trials for Experiment 1, so it was excluded from the dataset.

**Experimental Data**

**Table S2.**

| **Name** | **Condition** | **First Set of 60 Trials,**  **Blocks 1-4** | | | | **First Set Totals** | **Second Set of 60 Trials, Blocks 5-8** | | | | **Second Set Totals** |
| --- | --- | --- | --- | --- | --- | --- | --- | --- | --- | --- | --- |
| Blofeld | Parallel | 5/5 | 5/5 | 5/5 | 5/5 | 20/20 | 5/5 | 5/5 | 5/5 | 5/5 | 20/20 |
|  | Split | 1/5 | 3/5 | 3/5 | 3/5 | 10/20 | 2/5 | 2/5 | 4/5 | 4/5 | 12/20 |
|  | Crossed | 2/5 | 4/5 | 1/5 | 3/5 | 10/20 | 3/5 | 3/5 | 3/5 | 1/5 | 10/20 |
| Bruce | Parallel | 5/5 | 5/5 | 5/5 | 5/5 | 20/20 | N/A | | | | |
|  | Split | 3/5 | 0/5 | 1/5 | 1/5 | 5/20 |  |  |  |  |  |
|  | Crossed | 4/5 | 5/5 | 5/5 | 5/5 | 19/20 |  |  |  |  |  |
| Cheeky | Parallel | 5/5 | 5/5 | 5/5 | 4/5 | 19/20 | N/A | | | | |
|  | Split | 2/5 | 0/5 | 0/5 | 1/5 | 3/20 |  |  |  |  |  |
|  | Crossed | 5/5 | 3/5 | 3/5 | 5/5 | 16/20 |  |  |  |  |  |
| Harley Quinn | Parallel | 4/5 | 5/5 | 5/5 | 5/5 | 19/20 | *Excluded* | | | | |
|  | Split | 5/5 | 5/5 | 5/5 | 5/5 | 20/20 |  |  |  |  |  |
|  | Crossed | 2/5 | 1/5 | 2/5 | 1/5 | 6/20 |  |  |  |  |  |
| Loki | Parallel | 5/5 | 5/5 | 5/5 | 5/5 | 20/20 | 5/5 | 5/5 | 5/5 | 5/5 | 20/20 |
|  | Split | 1/5 | 1/5 | 3/5 | 4/5 | 9/20 | 2/5 | 2/5 | 2/5 | 2/5 | 8/20 |
|  | Crossed | 4/5 | 2/5 | 4/5 | 2/5 | 12/20 | 5/5 | 4/5 | 5/5 | 5/5 | 19/20 |
| Moriarty | Parallel | 5/5 | 3/5 | 4/5 | 5/5 | 17/20 | 3/5 | 4/5 | 5/5 | 3/5 | 15/20 |
|  | Split | 4/5 | 3/5 | 4/5 | 3/5 | 14/20 | 5/5 | 5/5 | 5/5 | 4/5 | 19/20 |
|  | Crossed | 2/5 | 2/5 | 3/5 | 2/5 | 9/20 | 1/5 | 4/5 | 1/5 | 3/5 | 9/20 |
| Neo | Parallel | 5/5 | 5/5 | 5/5 | 4/5 | 19/20 | 5/5 | 5/5 | 5/5 | 5/5 | 20/20 |
|  | Split | 4/5 | 2/5 | 0/5 | 0/5 | 6/20 | 1/5 | 0/5 | 0/5 | 4/5 | 5/20 |
|  | Crossed | 3/5 | 4/5 | 3/5 | 4/5 | 14/20 | 5/5 | 4/5 | 5/5 | 5/5 | 19/20 |
| Plankton | Parallel | 4/5 | 5/5 | 4/5 | 5/5 | 18/20 | 4/5 | 5/5 | 5/5 | 5/5 | 20/20 |
|  | Split | 3/5 | 5/5 | 2/5 | 3/5 | 13/20 | 5/5 | 2/5 | 4/5 | 3/5 | 14/20 |
|  | Crossed | 1/5 | 0/5 | 3/5 | 4/5 | 8/20 | 1/5 | 2/5 | 2/5 | 2/5 | 7/20 |
| Taz | Parallel | 5/5 | 5/5 | 5/5 | 5/5 | 20/20 | 5/5 | 5/5 | 5/5 | 5/5 | 20/20 |
|  | Split | 4/5 | 2/5 | 2/5 | 3/5 | 11/20 | 1/5 | 2/5 | 3/5 | 4/5 | 10/20 |
|  | Crossed | 2/5 | 4/5 | 3/5 | 2/5 | 11/20 | 3/5 | 5/5 | 2/5 | 1/5 | 11/20 |
| Spike | Parallel | 5/5 | 4/5 | 5/5 | 5/5 | 19/20 | *Excluded* | | | | |
|  | Split | 5/5 | 4/5 | 5/5 | 4/5 | 18/20 |  |  |  |  |  |
|  | Crossed | 0/5 | 2/5 | 0/5 | 1/5 | 3/20 |  |  |  |  |  |

***Table S2.*** Raw data for all individuals and all trial blocks in Experiment 1.

**Table S3.**

| **Name** | **Condition** | **Trajectory Prediction Trials** | | | | **Performance by Trial Type** | **Control  Condition Trials** | | | | **Performance by Trial Type** |
| --- | --- | --- | --- | --- | --- | --- | --- | --- | --- | --- | --- |
| Blofeld | ←^T^ | 2/5 | 4/5 | 4/5 | 3/5 | 13/20 | 5/5 | 5/5 | 5/5 | 5/5 | 20/20 |
|  | ↙ | 5/5 | 4/5 | 3/5 | 5/5 | 17/20 | 4/5 | 3/5 | 3/5 | 5/5 | 15/20 |
|  | ↖ | 4/5 | 3/5 | 5/5 | 5/5 | 17/20 | 5/5 | 5/5 | 5/5 | 5/5 | 20/20 |
|  | ←_B_ | 3/5 | 5/5 | 5/5 | 5/5 | 18/20 | 4/5 | 5/5 | 3/5 | 5/5 | 17/20 |
| **Performance in Each Block /20** | | **14** | **16** | **17** | **18** |  | **18** | **18** | **16** | **20** |  |
| Bruce | ←^T^ | 3/5 | 4/5 | 4/5 | 4/5 | 15/20 | 5/5 | 5/5 | 5/5 | 5/5 | 20/20 |
|  | ↙ | 4/5 | 5/5 | 4/5 | 5/5 | 18/20 | 5/5 | 4/5 | 5/5 | 4/5 | 18/20 |
|  | ↖ | 5/5 | 4/5 | 4/5 | 4/5 | 17/20 | 5/5 | 5/5 | 5/5 | 5/5 | 20/20 |
|  | ←_B_ | 5/5 | 4/5 | 5/5 | 5/5 | 19/20 | 3/5 | 5/5 | 5/5 | 5/5 | 18/20 |
| **Performance in Each Block /20** | | **17** | **17** | **17** | **18** |  | **18** | **19** | **20** | **19** |  |
| Loki | ←^T^ | 3/5 | 3/5 | 2/5 | 1/5 | 9/20 | 5/5 | 5/5 | 5/5 | 5/5 | 20/20 |
|  | ↙ | 5/5 | 5/5 | 5/5 | 5/5 | 20/20 | 5/5 | 5/5 | 4/5 | 5/5 | 19/20 |
|  | ↖ | 3/5 | 4/5 | 4/5 | 3/5 | 14/20 | 5/5 | 5/5 | 5/5 | 5/5 | 20/20 |
|  | ←_B_ | 5/5 | 4/5 | 5/5 | 5/5 | 19/20 | 4/5 | 4/5 | 2/5 | 4/5 | 14/20 |
| **Performance in Each Block /20** | | **16** | **16** | **16** | **14** |  | **19** | **19** | **16** | **19** |  |
| Moriarty | ←^T^ | 5/5 | 2/5 | 3/5 | 2/5 | 12/20 | 5/5 | 5/5 | 5/5 | 5/5 | 20/20 |
|  | ↙ | 3/5 | 5/5 | 5/5 | 5/5 | 18/20 | 5/5 | 5/5 | 5/5 | 5/5 | 20/20 |
|  | ↖ | 5/5 | 3/5 | 3/5 | 0/0 | 11/20 | 5/5 | 5/5 | 5/5 | 5/5 | 20/20 |
|  | ←_B_ | 3/5 | 2/5 | 3/5 | 2/5 | 10/20 | 4/5 | 5/5 | 5/5 | 5/5 | 19/20 |
| **Performance in Each Block /20** | | **16** | **12** | **14** | **9** |  | **19** | **20** | **20** | **20** |  |
| Neo | ←^T^ | 3/5 | 5/5 | 5/5 | 2/5 | 15/20 | 5/5 | 5/5 | 5/5 | 5/5 | 20/20 |
|  | ↙ | 5/5 | 5/5 | 4/5 | 5/5 | 19/20 | 3/5 | 3/5 | 5/5 | 5/5 | 16/20 |
|  | ↖ | 4/5 | 5/5 | 5/5 | 4/5 | 18/20 | 5/5 | 5/5 | 5/5 | 5/5 | 20/20 |
|  | ←_B_ | 5/5 | 5/5 | 5/5 | 5/5 | 20/20 | 4/5 | 3/5 | 5/5 | 5/5 | 17/20 |
| **Performance in Each Block /20** | | **17** | **20** | **19** | **16** |  | **17** | **16** | **20** | **20** |  |
| Plankton | ←^T^ | 1/5 | 2/5 | 1/5 | 3/5 | 7/20 | 5/5 | 5/5 | 5/5 | 5/5 | 20/20 |
|  | ↙ | 5/5 | 5/5 | 5/5 | 4/5 | 19/20 | 3/5 | 5/5 | 4/5 | 4/5 | 16/20 |
|  | ↖ | 2/5 | 1/5 | 3/5 | 2/5 | 8/20 | 5/5 | 5/5 | 5/5 | 5/5 | 20/20 |
|  | ←_B_ | 5/5 | 5/5 | 5/5 | 5/5 | 20/20 | 3/5 | 5/5 | 4/5 | 5/5 | 17/20 |
| **Performance in Each Block /20** | | **13** | **13** | **14** | **14** |  | **16** | **20** | **18** | **19** |  |
| Taz | ←^T^ | 2/5 | 5/5 | 5/5 | 4/5 | 16/20 | 5/5 | 5/5 | 5/5 | 5/5 | 20/20 |
|  | ↙ | 1/5 | 4/5 | 2/5 | 3/5 | 10/20 | 5/5 | 5/5 | 4/5 | 5/5 | 19/20 |
|  | ↖ | 4/5 | 4/5 | 5/5 | 3/5 | 16/20 | 5/5 | 5/5 | 5/5 | 5/5 | 20/20 |
|  | ←_B_ | 3/5 | 5/5 | 2/5 | 5/5 | 15/20 | 5/5 | 5/5 | 5/5 | 5/5 | 20/20 |
| **Performance in Each Block /20** | | **10** | **18** | **14** | **15** |  | **20** | **20** | **19** | **20** |  |

***Table S3.*** Raw data for all individuals and all trial blocks in Experiment 3. Arrow symbols denote the trajectory type, (a) top horizontal ←^T^, (b) top-bottom diagonal ↙, (c) bottom-top diagonal ↖, (d) bottom horizontal ←_B_.
